# Epidemiology of operation-related medical errors in inpatients in Japan: the JET study

**DOI:** 10.1186/cc14578

**Published:** 2015-03-16

**Authors:** Y Ohta, M Sakuma, D Bates, T Morimoto

**Affiliations:** 1 Hyogo College of Medicine, Hyogo, Japan; 2 Brigham and Women's Hospital and Harvard Medical School, Boston, MA, USA

## Introduction

Operation therapy is more invasive than medication therapy and then operation-related medical errors (MEs) might be of more significant impact than medication errors. We assessed the incidence and characteristics of operation-related MEs to improve patient safety in such patients.

## Methods

The Japan Adverse Event (JET) study was a prospective cohort study which had evaluated AEs and MEs at two tertiary care hospitals. We included all adult patients aged ≥15 years old who had operations over a 2-month period. The primary outcome of this study was the operation-related MEs, defined as any deviation from appropriate process of an operation or perioperative care. Trained nurses placed at each participating hospital reviewed all charts daily on weekdays, along with laboratories, incident reports, and prescription queries to collect any potential event. They also collected the characteristics of the patients in the cohort. Some operation-related MEs are associated with operation-related AEs, which are operation-related preventable AEs. After those suspected events were collected, physician reviewers independently evaluated them and classified them as operation-related MEs, AEs, or rule violations. Physician reviewers assessed and rated operation-related AEs according to the symptom and the severity of injury.

## Results

This study included 389 patients with 6,624 patient-days. The median age of patients was 69 years and 224 (58%) were male. Among these 389 patients, 31 patients had 46 operation-related MEs during their hospital stay and the incidence of operation-related MEs was 12 per 100 patients. Operation-related AEs occurred in 29 patients with 43 events. The most frequent symptoms for operation-related MEs were skin (26%), bleeding (21%), and central nervous system (14%). Among 46 operation-related MEs, 43 (93%) were not intercepted, and they resulted in operation-related AEs that were considered as preventable operation-related AEs. Nine of preventable operation-related AEs (21%) were fatal or life-threatening: five were nerve injury during operation and stroke after neurosurgical operation, and one biliary peritonitis after gastrectomy and cholecystectomy, and tension pneumothorax after lung lobectomy, and two unexpected massive bleeding due to vessels injury.

## Conclusion

Ninety-three percent of operation-related MEs resulted in operation-related AEs and 21% of them resulted in life-threatening events. Prevention of operation-related MEs should improve the mortality of surgical patients.

